# Community pharmacy integration within the primary care pathway for people with long-term conditions: a focus group study of patients’, pharmacists’ and GPs’ experiences and expectations

**DOI:** 10.1186/s12875-019-0912-0

**Published:** 2019-02-08

**Authors:** Ali M. K. Hindi, Ellen I. Schafheutle, Sally Jacobs

**Affiliations:** 1 Centre for Pharmacy Workforce Studies, Division of Pharmacy and Optometry, The University of Manchester, Oxford Road, Manchester, M13 9PT UK; 2 School of Health Sciences, Faculty of Biology, Medicine and Health, The University of Manchester, Oxford Road, Manchester, M13 9PT UK

**Keywords:** Community pharmacy, Primary care, General practice, Long-term condition, Patients, Pharmacists, General practitioners, Integration, Collaboration

## Abstract

**Background:**

This study aimed to use marketing theory to examine the views of patients, pharmacists and general practitioners (GPs) on how community pharmacies are currently used and to identify how community pharmacy services may be better integrated within the primary care pathway for people with long-term conditions (LTCs).

**Methods:**

A qualitative research design was used. Two focus groups were conducted with respiratory patients (*n* = 6, 5) and two with type 2 diabetes patients (both *n* = 5). Two focus groups were held with pharmacists (*n* = 7, 5) and two with GPs (both n = 5). The “7Ps marketing mix” (“product”, “price”, “place”, “promotion”, “people”, “process”, “physical evidence”) was used to frame data collection and analysis. Data was analysed using thematic analysis.

**Results:**

Due to the access and convenience of community pharmacies (“place”), all stakeholder groups recommended using community pharmacies over GP practices for services such as management of minor ailments, medication reviews and routine check-ups for well managed LTCs (“product”). All stakeholder groups preferred pharmacy services with clear specifications which focused on specific interventions to reduce variability in service delivery and quality (“process”). However, all stressed the importance of having an appropriate system to share relevant information, allowing pharmacists and GPs two-way flow (“process”). Pharmacists and GPs mentioned difficulties in collaborating with each other due to inter-professional tensions arising from funding conflicts, which leads to duplication of services and inefficient workflow within the primary care pathway (“people”). Patients and GPs were sometimes doubtful of community pharmacies’ potential to expand services due to limited space, size and poor quality consultation rooms (“physical evidence”). However, all stakeholder groups recommended promoting community pharmacy services locally and nationally (“promotion”). Patients felt the most effective form of promotion was first-hand experience of high quality pharmacy services and peer word-of-mouth. The added value of using pharmacy services was faster access and convenience for patients, and freeing up GPs’ time to focus on more complex patients (“value”).

**Conclusions:**

Using the 7Ps marketing mix highlighted factors which could influence utilisation and integration of community pharmacy services within the primary care pathway for patients with LTCs. Further research is needed to identify their relative importance.

**Electronic supplementary material:**

The online version of this article (10.1186/s12875-019-0912-0) contains supplementary material, which is available to authorized users.

## Background

Healthcare organisations worldwide are under substantial pressure from increasing patient demand [1]. In the United Kingdom (UK), this has led to shifting many secondary care activities towards primary care and increasing workload pressures on general practitioners (GPs) [2, 3]. The increasing population of patients with long-term conditions (LTCs) are associated with high levels of morbidity, healthcare costs and GP workloads [4–6]. These patients present with a range of healthcare needs such as regular monitoring of condition(s), management of complex dosing regimens, ensuring appropriate use of medications and lifestyle education [7, 8].

Policy-makers worldwide have recognised the potential of community pharmacies to meet some needs of patients with LTCs and reduce workload pressures on GPs [9–12]. Community pharmacies are accessible and convenient primary care venues with long opening hours and non-appointment-based services [9, 10]. Community pharmacists are increasingly clinically trained healthcare professionals whose skills and knowledge could be further utilised [7, 11, 13]. International health policy initiatives have focused on extending community pharmacy services through novel reimbursement structures to help alleviate existing pressures in general practice [14].

The UK National Health Service (NHS) introduced new community pharmacy contractual frameworks in England and Wales in 2005 and Scotland in 2006, which reimburse pharmacists for clinical, medicines and public health services, in addition to medicines supply (i.e. dispensing) [9]. In England, the contractual framework composes of three service types: “essential”, “advanced” and “locally commissioned”. “Essential services” cover traditional services provided by all community pharmacies (dispensing medications/appliances, repeat dispensing, signposting i.e. informing or advising people to visit other health/social care providers and support organisations, when appropriate). “Advanced services” focus on medication reviews conducted by pharmacists as well as flu vaccinations and urgent medicines supply. The two main medicines review services are the Medicines Use Review (MUR) and the New Medicine Service (NMS). Both services focus on improving medication understanding and adherence for patients with LTCs [15]. Similar services also exist in Wales [16], Northern Ireland [17] and Scotland [18]. To preserve patient privacy and confidentiality, consultation rooms became a prerequisite for community pharmacies offering advanced services under the new contract. There are also other medication and public health services which can be commissioned according to local need. These “Locally commissioned services” include minor ailments management, lifestyle advice, blood pressure checks, cholesterol tests and smoking cessation services. These extended services currently provide opportunities for community pharmacists to offer support for patients with LTCs that extends beyond medicines supply.

Despite the new community pharmacy contractual frameworks in the UK, there have been barriers to pharmacists providing extended services such as inadequate resources, time constraints, unsuitable premises and lack of management support [11, 19–21]. There is also evidence that patient awareness, demand and uptake of community pharmacy services are low [22–25] and community pharmacy integration within primary care has been slow [26]. The primary care pathway for patients with LTCs is the healthcare route these patients take for ongoing treatment and management of their conditions [4, 6]. GPs are central to this patient pathway, but community pharmacy services have traditionally been quite separate and GPs may not be aware or necessarily supportive of extended services due to concerns about pharmacists’ financial motives, competencies, and encroachment of professional boundaries [27]. This lack of GP support/awareness also impacts patients’ awareness, demand and use of community pharmacy services as many patients seek GPs endorsement for use of healthcare services [25, 26]. A lack of community pharmacy integration within this patient pathway prevents benefits to patients or the healthcare system through the optimal use of extended pharmacy services [27]. It is important to identify how community pharmacies could be better used and integrated within the patient’s primary care pathway, as effective collaboration between GPs and community pharmacists will be an important factor to optimise patient care [25, 26].

Recent UK policy initiatives have highlighted the need to further extend community pharmacy services and enhance integration within primary care [7, 10]. This requires better understanding of key stakeholders’ (patients’, pharmacists’, and GPs’) expectations, needs and preferences regarding the contribution of community pharmacy. Previous research has explored stakeholders’ perspectives of community pharmacy services [22, 23, 25, 28–30]. However, these studies focused on specific services, rather than the general expectations and awareness of the extended role of community pharmacies. Moreover, despite GP practices being central to the patient primary care pathway, studies rarely consider the influence that GPs have on patients accessing community pharmacy services, nor do they explore GPs’ expectations of community pharmacy services in relation to services they currently deliver.

There has been growing interest from public sector organisations in the application of marketing theories to enhance service provision to achieve organisational goals [31, 32]. The driver being that marketing theories focus on identifying consumer (and other stakeholder) needs and preferences whilst considering other organisational complexities [31, 32]. It has been argued that the use of marketing theories in public sectors could provide a better understanding of how these organisations could meet the expectations of their target population [31, 33]. Moreover, previous studies have demonstrated the applicability of marketing theories to shed light on factors which influence the demand and uptake of public sector services [34, 35]. Therefore, marketing theories may be applied to primary healthcare and, specifically, the community pharmacy context to provide valuable insights such as: identifying stakeholders’ needs and preferences, understanding factors that influence service uptake, and exploring how services could meet quality standards [36–38]. Despite the wide recognition that marketing theories are the cornerstone for successful implementation of new products or services [36, 39], marketing theory has had limited application in community pharmacy research [36, 38–40].

**Fig. 1:**
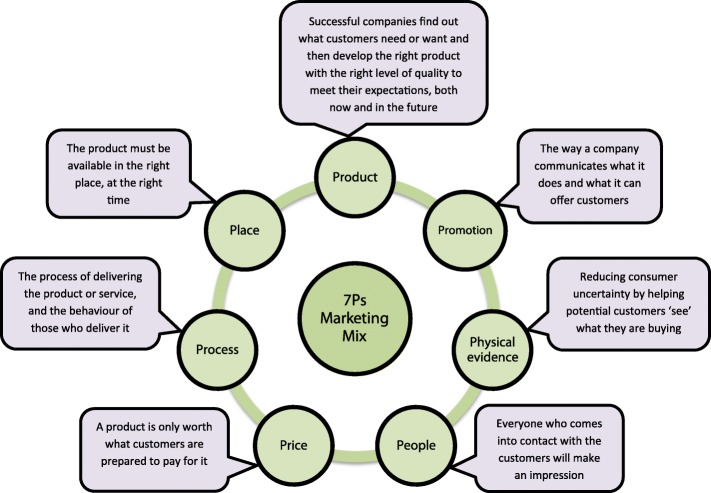
7Ps marketing mix proposed by Booms and Bitner

The “7Ps marketing mix” which was used in this study has been considered as one of the cornerstones of marketing theory [32, 41]. The 7Ps consists of seven components (“*product”, “price”, “place”, “promotion”, “people”, “process” and “physical evidence”)* (Fig. 1) that an organisation should account for to successfully market their product or service to target customers [41]. The 7Ps are based on understanding what consumers want/need from a service whilst accounting for the influence of service design, service delivery and external communications on consumers’ perceptions of services. Evidence has shown that the 7Ps can be applied to organisations providing public services [31, 36, 42]. Moreover, two studies demonstrated the influence of 7Ps on patients’ accessing and utilising hospitals [43, 44].

The aim of this study was to use marketing theory (7Ps marketing mix) to explore how community pharmacies in the UK are currently used and to identify how their services may be better used and integrated within the primary care pathway for people with LTCs.

## Methods

### Study design and setting

A qualitative research design was used. Separate focus groups were conducted to explore the views of stakeholders, i.e. patients with LTCs, pharmacists and GPs. The study was set in Greater Manchester, England.

### Theoretical framework

The “7Ps marketing mix” was used to frame data collection and analysis. The 7Ps was applied here in relation to community pharmacy services (Table 1), informed by findings from an earlier systematic review [22].

**Table 1:** 7Ps marketing mix components in relation to community pharmacy services within the primary care pathway for patients with long-term conditions

Product	Exploring stakeholders’ expectations and perceptions of community pharmacy services within the patient primary care pathway.
Process	Exploring stakeholders’ expectations and experiences regarding utilisation and delivery of community pharmacy services.
People	Exploring how interactions between stakeholders affect perceptions and delivery of community pharmacy services.
Place	Exploring access to community pharmacies
Physical evidence	Identifying how physical characteristics of community pharmacies influence expectations and perceptions of stakeholders
Promotion	Investigating how community pharmacy services are communicated and promoted
Price	Investigating what added value stakeholders place on community pharmacy services within the primary care pathway

### Sampling

Purposive criterion sampling was used to recruit study participants [45].The characteristics patients were selected on were that they had one or more of the common long-term conditions: type 2 diabetes, asthma, chronic obstructive pulmonary disease (COPD). Many community pharmacy services already exist which are relevant to patients with these conditions such as medication reviews, health checks (blood pressure, cholesterol tests etc.), influenza vaccinations and smoking cessation [46–50]. Community pharmacists were recruited based on experience offering extended pharmacy services. There were no specific characteristics set for recruitment of GPs.

Two focus groups were conducted for each: patients with diabetes, patients with respiratory conditions, pharmacists, and GPs. Based on expert recommendations, this sample was deemed sufficient to meet the aims of this study [51–53].

### Recruitment

Patients were identified through two patient charity organisations and two NHS-supported online resources involving patients and members of the public in research. The research team provided study information for dissemination with contact details (invitation letters, participation information sheets and/or participation flyers). Patients who contacted the research team were invited to take part via phone/email.

Pharmacists were identified through existing networks. Known contacts, the Greater Manchester Local Pharmaceutical Committee and Greater Manchester Clinical Commissioning Groups were asked to circulate study information to pharmacists and GPs respectively. Pharmacists/GPs who contacted the research team were invited to take part by phone/email. Pharmacists and GPs were also identified and recruited through advertising on social media. The research team was unable to identify how many participants refused to participate due to these recruitment methods. Prior to study commencement, the research team had no established relationship with participants. All participants were reimbursed for their time and reasonable travel expenses.

### Data collection

The development of the focus group topic guides was informed by the 7Ps marketing mix framework and existing literature on the topic [22]. Each marketing mix component (“P”) was used to frame questions relative to participants’ experiences and expectations of community pharmacy services. As prompts, a list of community pharmacy services was provided for participants during the focus groups (Table 2).The topic guide differed somewhat for patients, pharmacists and GPs, to account for their different roles within primary care (Additional file 1). The pharmacist topic guide was tested in a pilot focus group with university staff who had experience working in community pharmacies. Following the pilot, participants were asked for feedback and final revisions made. The lead author received considerable training to conduct focus groups (i.e. courses, workshops, focus group pilot) and was supported by both co-authors who are both experienced qualitative researchers and co-facilitated all groups.

**Table 2:** List of services community pharmacies offer

Medicines related services	Public health services
• Medicines Use Review	• NHS Health checks
• New Medicine Service	• Asthma management (support)
• Minor Ailments scheme	• Diabetes management (support)
• Asthma inhaler technique service	• Flu vaccination
• COPD rescue packs toolkit	• Travel health
• Repeat dispensing	• Smoking cessation
• Independent prescribing by pharmacists	• Weight management
• Out of hours (access to medicines)/ emergency supply	• Alcohol screening and brief advice
• Domiciliary/ Home care support	• Emergency hormonal contraceptive

Most focus groups were conducted at The University of Manchester; only one GP group was conducted at a GP surgery conference room, between January and April 2018. The focus groups were facilitated by the first author and co-facilitated by one of the co-authors, who took handwritten notes. Each focus group lasted between 50 and 110 min. After each focus group, a debrief session was held between the facilitators to discuss and summarise key points.

### Data analysis

All focus groups were audio-recorded with verbal and written consent and transcribed verbatim. Transcriptions were imported into NVivo11 to manage the data analysis process [54]. Data analysis was iterative, commencing after the first focus group, with transcripts analysed using thematic analysis [55]. This method provided rich detailed descriptions of the dataset under themes identified using both inductive and deductive approaches, arising from the data itself whilst mapping onto the 7Ps marketing mix model. The first author followed the six phases of thematic analysis: familiarisation with the data, generating initial codes, searching for themes, reviewing themes, defining and naming themes, and producing the report [55]. Analysis and themes were discussed with the co-authors in regular meetings throughout analysis.

## Results

A total of 43 participants took part in eight focus groups. Two focus groups were conducted with asthma/COPD patients (*n* = 6 and 5), two with type 2 diabetes patients (both *n* = 5). Two focus groups were held with pharmacists (*n* = 7 and 5) and two with GPs (both *n* = 5). Participant characteristics are provided in Table 3.

**Table 3:** Participant demographics

Focus group	Participants	Participant Number	Gender
1	Asthma/COPD patients	1	F
		2	F
		3	F
		4	M
		5	F
		6	M
2	Asthma/COPD patients	7	M
		8	F
		9	F
		10	F
		11	F
3	Diabetes patients	12	M
		13	F
		14	M
		15	M
		16	F
4	Diabetes patients	17	F
		18	M
		19	M
		20	M
		21	M
5	Pharmacists	22	F
		23	M
		24	M
		25	M
		26	M
6	Pharmacists	27	M
		28	F
		29	M
		30	F
		31	F
		32	F
		33	F
7	GPs	34	F
		35	M
		36	M
		37	F
		38	M
8	GPs	39	F
		40	M
		41	M
		42	F
		43	M

**Fig. 2:**
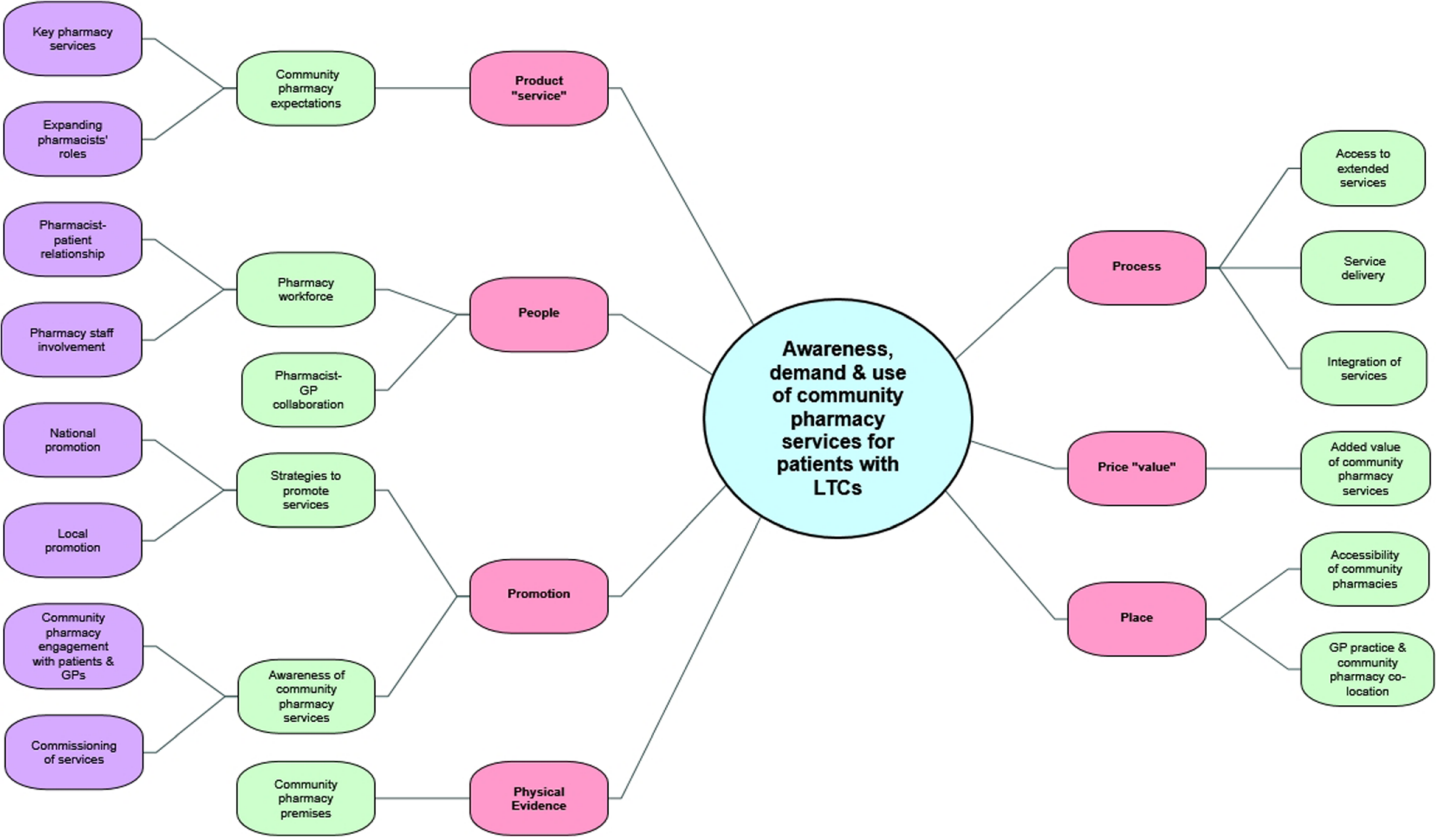
Conceptual framework developed from mapping themes/subthemes onto 7Ps marketing mix components

Emerging themes were mapped onto the 7P components to develop a conceptual framework (Fig. 2). This identified factors influencing awareness, demand and use of community pharmacy services for patients with LTCs. Findings are presented together, whilst identifying which of the three stakeholder groups they stemmed from, thus similarities and key differences between them.

## Product

### Community pharmacy expectations

All groups of stakeholders discussed their expectations of community pharmacy services within the primary care pathway. These expectations were centred on “key pharmacy services” and “expanding pharmacists’ roles”.

### Key pharmacy services

All stakeholders’ expectations of community pharmacy services were based on how patients used community pharmacies for their LTCs. All stakeholder groups’ expectations of community pharmacies over and above traditional supply functions were predominantly to provide medicines management for patients with LTCs. This involved ensuring appropriate medication usage, educating patients on their medications, double-checking prescriptions and referring patients to GPs if necessary. Public health services were not usually discussed by any of the stakeholder groups. Even when stakeholder groups were probed about public health services, all stakeholders mentioned that patients rarely used them and were unaware of them being offered by community pharmacies. Moreover, participants in all focus groups considered public health services to be expanded rather than standard community pharmacy services.

*“Being able to talk about the medicines and the side-effects and any worries or concerns that I’ve got and anything associated with it. That’s the main thing to me, ‘cause that’s the basis of how a pharmacist used to be”.* [F1, asthma/COPD patient FG1]

All focus groups saw the potential for using community pharmacies to reduce GP visits. Most diabetes patients had experienced difficulties in obtaining GP appointments for regular check-ups/procedures for well-managed conditions (e.g. blood tests) and recommended community pharmacies to deliver such services. Some asthma/COPD patients were aware that community pharmacies offered inhaler technique services but proposed community pharmacies routinely provide inhaler technique and nebuliser services due to difficulties accessing GP services. Similarly, some GPs suggested community pharmacies could provide regular check-ups and medication reviews for patients with well-managed LTCs to reduce their workload pressures. Overall, there was a shared agreement between all focus groups that patients with LTCs would benefit from community pharmacies regularly providing check-ups and medication reviews.

*“I have to have blood tests and then I wait ten days and another appointment and it's clogging up their [GPs] resources for just managing a condition that's not changing. Admittedly, if it changes, the levels change, then go to the doctor, but for someone like me that it hasn't changed in ten years, it would probably be more efficient for that diabetic management service to actually do what the GP does for me.”* [M15, diabetes patient FG3]

On the other hand, all groups of stakeholders perceived GP practices to be more suitable than community pharmacies for management of patients’ conditions which required a range of clinical interventions such as diagnosis of symptoms, physical examinations and alteration of medications. GPs and practice nurses were viewed by most patients and all GPs as more experienced and authoritative healthcare professionals to manage patients’ conditions and perform clinical services. Only a few patients in the respiratory and diabetes focus groups argued that pharmacists were well-suited to manage patients’ conditions and perform clinical services. All pharmacists also believed that patients would be more comfortable with GPs and practice nurses managing their conditions and performing clinical services.

*“I’d rather see him [GP] when I’m thinking of changing medication …because he knows sort of all different episodes I’ve had so…I think for things like physical health because you may have been to your GP several times for different aspects, then I think it’s better to see them for that”.* [F8, asthma/COPD patient FG2]

### Expanding pharmacists’ roles

All stakeholder groups perceived pharmacists’ skills to be underutilised and supported more active involvement in patients’ LTCs. Patients and pharmacists were generally more confident than GPs in pharmacists’ abilities to expand their roles. Most patients were supportive of pharmacists providing minimally invasive procedures, such as blood tests. Similarly, most GPs supported pharmacists providing minimally invasive procedures but needed to be assured that the pharmacists providing these services were competent.

*“I'm just wondering if there is just a small leap, sort of like an additional professional training that would allow pharmacists who are trained medical people to be able to take bloods and maybe offer the services that they do as side lines.”* [M14, diabetes patient FG3]

Conversely, pharmacists underestimated patients’ support for them and perceived that they preferred GPs and nurses to manage their LTCs.

*“But I think people's expectation is that the GP and the nurse manages their diabetes and that's not really much to do with pharmacy”.* [M24, pharmacist FG5]

Some patients and GPs were indeed critical of pharmacists, indicating that they lacked determination to take more responsibility. They expressed a need for pharmacists to be more active in expanding their roles and to distinguish themselves from other pharmacy staff. Pharmacists also acknowledged that not all pharmacists were willing to expand their roles and face new challenges. The general consensus amongst all stakeholder groups was that, unless pharmacists took the initiative, their perceived status as “*shopkeepers*” would not change.

*“But what are they [pharmacists] doing? Because they should be doing a lot more than they’re being paid to do or whatever, they’re running a business to sell their shampoos and whatever, but there must be something else that we could be doing to really enhance that role. So why aren’t they coming out?”* [M36, GP FG7]

## Process

### Access to extended services

There was little indication by patient and pharmacist focus groups that patients with LTCs sought out extended services. Rather, patients obtained access through active recruitment by pharmacy staff at the counter. None of the patients recalled GPs referring them to pharmacy services. Similarly, most GPs did not recall referring patients to any pharmacy services. Both patients and GPs held pharmacists partially responsible for this lack of GP referrals, believing pharmacists did not promote their skills and services beyond dispensing.

*“It [accessing a pharmacy service] will depend on the people who sell it…how much further they are prepared to go that extra half mile by telling you or helping you with things, or giving you advice”.* [F5, asthma/COPD patient FG1]

### Service delivery

Both pharmacists and GPs suggested that some extended services lacked clear specifications, leading to disparities in service delivery and quality. Some GPs’ mentioned that they were less likely to refer their patients to community pharmacy services which they believed lacked clear specifications.

*“I'm probably not gonna actually advise someone to go to a community pharmacist weight management, because I just don't know what they do and how useful it is”.* [M40, GP FG8]

Most pharmacists and GPs proposed developing community pharmacy services that had clear specifications and focused on a single, specific intervention, mentioning flu vaccination and inhaler technique services as examples. Pharmacists believed that such services would enhance consistency in service delivery and quality as they were easier for pharmacists to deliver and promote to patients and other healthcare providers. All of the patients in the respiratory and diabetes focus groups similarly expressed preferences for community pharmacy services that focused on one particular intervention such as cholesterol and blood tests emphasising that procedures for these services were easy for them to understand.

*“They need to be specific as well. If the service is well designed, well explained and you’re just focusing on one particular problem, one specific thing that you can everybody fully understands, you know, what is it all about, then you can well…easily communicate that with a doctor, patient, yourself”.* [M26, pharmacist FG5]

Pharmacists and GPs also perceived that inadequate time, management pressures to perform services and pharmacy managers’ financial conflicts of interest negatively influenced the quality of community pharmacy services. Some patients in the respiratory and diabetes focus groups were wary of pharmacists’ workload pressures and doubted their capacity to provide extra services beyond dispensing medications. When discussed, all stakeholder groups generally agreed that pharmacists’ workload was a major barrier to providing extra services beyond dispensing medications.

*“I was thinking to myself, if they’re so stretched and they’re so busy and they’re sometimes making mistakes with prescriptions, there’s a chain of pharmacies, how are they going to offer all these things if they’re struggling to just do the medication, you know?”* [F10, asthma/COPD patient FG2]

### Integration of services

It was evident from all focus group discussions that community pharmacies and GP practices needed to improve integration to establish a seamless pathway for patients with LTCs. All stakeholder groups agreed that they currently worked in separate siloes which led to duplication of services and miscommunication between pharmacists and GPs.

*“You’ve got to address the problem of duplication of effort as well, it’s not a seamless setup, is it, they've not really thought through the process”.* [F30, pharmacist FG6]

Some pharmacists and GPs suggested community pharmacies should provide services that were not widely offered by GPs such as domiciliary support and medication reconciliation. All GPs in one of the focus groups also discussed including community pharmacies as part of care plans for patients with LTCs. GPs felt that having a shared care plan would significantly enhance teamwork between community pharmacy and GP practice staff. GPs also believed that GP practices would be more likely to collaborate with community pharmacies if they had shared care plans.

*“I would say that there hopefully would be some agreement about when that patient needs a routine review, who does it, what particular things need to be checked, the action plan, and it would be a standard plan that everybody’s got… I think that could be applied to several different long-term conditions cause you’d have the buy-in then of the practice”.* [F37, GP FG7]

All stakeholder groups believed pharmacists required more access to patient information (i.e. medical records) to have a better overall understanding of patients’ conditions. Lacking full patient information made pharmacists hesitant in clinical decision-making for fear of making mistakes. GPs were similarly concerned with the safety implications of pharmacists providing extra services without having access to patients’ medical records and it also reduced patients’ confidence.

*“We’ve not got the full picture. Certainly with me, you know I will think twice about, do I really want to go down this process, because I don’t know where it's going to lead and I might find myself unable to give the right answers or not be confident that what I've said is correct in light of information that I don't know”.* [M23, pharmacist FG5]

Conversely, some pharmacists were concerned that being granted more access to patient information could hold them more accountable for patient outcomes. Hence, some suggested restricting pharmacists’ access to specific services which required additional patient information. All GPs and patients suggested restricting pharmacy access to medical records to maintain patient confidentiality and were mainly concerned about non-healthcare staff accessing medical records.

*“So if they could have obviously not the whole patient record, but if they could have access to part of it, mainly the meds and the bloods, would work. Because it's patient confidentiality issues, isn’t it?”.* [F39, GP FG8]

Nonetheless, all focus groups emphasised the importance of having a shared information system to facilitate collaborative working. Pharmacists and GPs referred to current difficulties for GPs to embed community pharmacy services: referring patients to community pharmacy services was considered *“unsafe”* by GPs as patient interactions and outcomes were not documented and communicated to them.

*“But we don’t really know what interactions are occurring until the patients come back in and tell us that I saw A, B, C or D healthcare professional. So ideally to back all of this up, we need an integrated IT system and we haven’t got one. Then we could do a lot more if we did. Otherwise there are gaps in information”.* [M35, GP FG7]

### People

#### Pharmacy workforce

##### Pharmacist-patient relationship

Patients in all focus groups often referred to their relationship with their pharmacists and how it influenced their perceptions of community pharmacies. There was agreement amongst pharmacist and patient focus groups that patients were more willing to accept extended services if they had good relationships with the pharmacist. They believed that continuity of care with the pharmacist added a *“personal touch”* to services provided which enhanced their uptake. However, all focus groups perceived patients with LTCs to be more familiar with their GPs and practice nurses than with their pharmacists. They mentioned that unlike GP practice staff, community pharmacists changed regularly. Therefore, GP practice staff offered more continuity in provision of services which was seen as an important factor for managing long-term conditions.

*“Yeah, and of course… there isn’t that constant workforce within pharmacies also, so they come and go, and that’s a problem”.* [M36, GP FG7]

##### Pharmacy staff involvement

All pharmacists mentioned the importance of having the whole pharmacy team engaged with delivery of services in order to reduce workload pressures on pharmacists and enhance workflow. Pharmacists believed their staff played an important role in identifying and recruiting patients for extended services. Patient and GP focus groups did not discuss the importance of other pharmacy staff.

*“There is something about counter staff because things like new medicine service…I gave them all of the responsibility to get the patients in. But that was brilliant and then that responsibility shifted from me and it was then, the care of the patients was my focus”.* [M27, pharmacist FG6]

### Pharmacist-GP collaboration

All Pharmacists and GPs stressed the importance of enhancing inter-professional relationships to improve the delivery and uptake of services for patients with LTCs. Both referred to difficulties in collaborating due to tensions arising from funding conflicts, giving the example of influenza vaccination services, where GPs sometimes worked against pharmacists and advised patients to avoid using community pharmacies. Some patients in the respiratory focus groups were also aware of these funding conflicts which made them question collaboration between pharmacists and GPs. Regardless of funding conflict awareness, patients in all focus groups were in agreement that their pharmacists and GPs did not collaborate with each other.

*“I think it would be nice for us all to be working together but there are barriers, I think, they’re potential barriers. One, for example, is with the flu vaccine, the pharmacist is looking for their income and we’re looking for our income, so that has a sort of a point of contention”* [M35, GP FG7]

Pharmacists and GPs in all focus groups argued that unless both were adequately remunerated for joint working, they were unlikely to prioritise the promotion or provision of extended pharmacy services.

*“But I think realistically, this is more than sharing the love, it’s sharing the funding, because no one’s going to do this [extended pharmacy services] unless they have suitable recompense, so they’ll [pharmacists] be, yes, helping us out…but at the same time, they’re not gonna do it for free, are they?”.* [F37, GP FG7]

All of the pharmacists also discussed GPs’ unwillingness to recognise pharmacists as healthcare providers as another barrier to collaboration. They believed GPs did not have an understanding of what pharmacists could offer to patients with LTCs. Conversely, all GPs often discussed community pharmacists’ potential to expand and become an integral part of patients’ primary care pathways. Moreover, GPs expressed interest in enhancing communication with pharmacists, preferring face-to-face communication over telephone/fax.

*“If they [community pharmacists] actually came into the surgery one day a week or something… because we could flag up any concerns or issues and we could discuss things there and then. So yeah, if they had direct contact once a week or something that would be quite handy”.* [M41, GP FG8]

A few pharmacists in both focus groups mentioned instances of successful collaboration when they had invested time and effort to communicate their roles and demonstrate their skills to GPs. When discussed, all pharmacists and GPs believed that although GPs were receptive to such approaches, they wanted pharmacists to be more proactive.

*“But then to be honest with you, when you try to go and try to advertise it [extended services], you're trying to go and make that relation happening, they see you as a useful point of contact for various reasons and where you can build on it”.* [M26, pharmacist FG5]

However, some pharmacists were cautious of getting more involved with GPs as negative outcomes could reduce their pharmacy’s revenue.

*“Because the danger of getting more involved and communicating more with GPs is if something goes wrong that's your pipeline of money threatened because GPs do influence where patients go. So, you might be savvy to just have a little bit of communication now and again”.* [M23, pharmacist FG5]

### Place

#### Accessibility of community pharmacies

Flexible opening hours and non-appointment based services were considered by all stakeholder groups to be community pharmacies’ greatest advantage over general practices. Due to the accessibility of community pharmacies, patients preferred them over GP practices for non-urgent and less invasive services.

*“But my biggest thing is like the simple stuff when you’re going for your six-monthly checks for your blood tests and your blood pressure, you're waiting for at least three weeks to get a doctor's appointment. So stuff like that can probably be done, I think, at a pharmacy”.* [M20, diabetes patient FG4]

Paradoxically, some pharmacists perceived this ease of access as a significant barrier to providing extended services as it increased workloads and tied them to medicines supply.

*“It’s quite difficult to deliver quality services on top of doing other stuff …because part of the advantage of community pharmacy is that people don’t need to book an appointment, but actually that makes it more difficult to plan into your workflow…”* [M24, pharmacist FG5]

#### GP practice and community pharmacy co-location

There were mixed opinions amongst patients and pharmacists on the importance of community pharmacies being located next to GP practices. GPs, however, were unanimous in their opinions that community pharmacies were better co-located in GP practices, which some patients also perceived to be more convenient. Both pharmacists and GPs perceived co-location to improve their communication, relationship and workflow.

*“But in the previous practice I was in, the pharmacist… it was a community pharmacy, it wasn’t the GP’s pharmacy, but it was co-located in the same building. And in that situation I knew all the pharmacists by face and I’d sometimes just pop in there to ask a question and they’d sometimes just pop in to ask me a question. So I had a much more solid understanding of what they did”.* [M40, GP FG8]

### Physical evidence

#### Community pharmacy premises

Patients and GPs were sometimes doubtful of community pharmacies’ potential to expand services for LTCs due to limited space and size. All stakeholder groups were in agreement that current community pharmacy premises gave the impression of a retail shop rather than a healthcare venue.

*“They have a shop and there’s a professional and there’s somebody counting pills and there’s somebody producing a prescription. Well that just doesn’t make sense in the twenty-first century… I think something more radical has to happen and we have to break down these kinds of physical walls”.* [M36, GP FG7]

In addition, all focus groups criticised the size and quality of consultation rooms and stressed the importance of having sufficient privacy within the pharmacy.

“M4: It’s only like a little cupboard, isn’t it?”

“F5: Yeah, a lot of them have a poky little room, yeah.”

[Asthma/COPD patients FG1]

### Promotion

#### Awareness of community pharmacy services

##### Community pharmacy engagement with patients and GPs

All patients were generally unaware of the considerable heterogeneity in community pharmacy types and organisations. Hence, patients and pharmacists believed that patients’ awareness of community pharmacy services was influenced by how active their usual pharmacy was at offering a range of services. Patients’ rarely or infrequently visiting community pharmacies were unaware of most services offered by their pharmacy. All GP participants’ awareness of community pharmacy services was mostly dependent on services delivered by community pharmacies near their GP practice.

*“If they [patients] go to one of the really engaged sort of right on the cusp of the profession, delivering lots of services, multiple consultation rooms, everything going there, then generally just because that's happening around them, they'll have much more exposure. If it's not happening and it is very much a supply shed style, then it's probably not so much”.* [M27, pharmacist FG5]

Patients and pharmacists both felt that patients needed to better engage with the community pharmacy to be aware of services offered. Pharmacists also stressed the need to incentivise GPs to refer patients to extended pharmacy services. GP participants were concerned that referring patients might affect GP practice revenue.

*“There's got to be benefit in it to a GP [to refer patients to community pharmacy services]. You’ve got to sell it on the basis that this is going to make your life easier and it’ll count towards your QOFs [quality and outcomes framework] if you do that. If it's a win-win then yes, but as [other participant] said, if it's a competitive thing, no”.* [M24, pharmacist FG5]

##### Commissioning of services

Both pharmacists and GPs were critical of the lack of consistency with which community pharmacy services were delivered. This was primarily due to differences in the commissioning of community pharmacy services within different localities. Hence, some pharmacists mentioned being unable to offer some services to patients which reduced patients’ awareness of services offered.

*“Especially because a lot of patients talk to each other, oh, you know, take your child to such and such a pharmacy you can get it free. But then when they turn up to that pharmacy and they say, sorry, we don't do it, they feel disappointed and that way, they might not bother next time to ask for any service”.* [F33, pharmacist FG6]

Pharmacists also highlighted that there were different service specifications within different commissioning areas which made it even more difficult for patients to know what extended services were offered. All stakeholder groups were in agreement that the variation in services offered amongst different community pharmacies blurred patients’ awareness of services offered.

*“And I said could you possibly check my blood pressure? Yeah, no problem, so I did it and it was fine, and everything seemed to just go back to normal, so I thought, hang on a minute, if it’s the pharmacists that won’t do can’t do it, why is one saying we can’t do it and one does say we can do it?”.* [F9, asthma/COPD patient FG2]

In addition, all GPs and pharmacists emphasised that service delivery was often dependent on individual pharmacists. Therefore, even if services were commissioned, patients who visited these pharmacies were not guaranteed to receive them. In addition, GPs in all focus groups did not want to risk referring patients to community pharmacies for services that may not be offered.

*“It’s very frustrating because the uptake from community pharmacists varies, and you send a patient off to this particular branch where you know they do it and the patient comes back after twenty minutes, oh, that pharmacist is not on today, you know? So, well, that was a wasted effort”.* [F37, GP FG7]

#### Strategies to promote services

##### Local promotion

Some patients suggested that pharmacists put leaflets in patients’ bags and use emails/texts/social media to promote services. Nonetheless, most patients in all focus groups felt that the most powerful form of service promotion would be experiencing extended pharmacy services first hand. In addition, pharmacists and patients discussed patients being more confident in the quality of services if recommended by peers with a good service experience (i.e. word-of-mouth). GPs made little mention of local promotion as they were mainly concerned with promoting community pharmacy services on a national level.

*“Do you know what the best thing's been for us this year being with the flu jab service is word-of-mouth. Oh, I went to Ken down the road, it was fantastic I didn't feel it. You had your flu jab, and then Ken's got a line of people outside his branch, because that one person's had a really good experience”.* [F28, pharmacist FG6]

##### National promotion

All stakeholder groups highlighted the importance of nationally promoting community pharmacy services to attain a wider level of public awareness. They commonly attributed the success of influenza vaccinations to its consistent national promotion through the media. In addition, some pharmacists mentioned educating patients that NHS community pharmacy services are free to them as some patients assumed all community pharmacy services charged a fee.

*“We can advertise all day long in our pharmacy, putting posters up, but unless someone’s going to come into the pharmacy and see those posters who's going to see it? So, if it's advertised further afield, on TV, radio, newspapers, and so on, that's going to get people to realise what can be done in a pharmacy”* [M25, pharmacist FG5]

All GPs stressed the importance of raising public awareness regarding the benefits of using community pharmacy services to reduce unnecessary patient visits to GPs.

*“So I think overall, I'm talking about a strategic level now in terms of informing and education, educating patients as to what services they can get, at the right time, at the right place and that would help a lot in reducing unnecessary visits to the GP”.* [M43, GP FG8]

### Price

#### Added value of community pharmacy services

Patient and GP focus groups discussed features which added value to obtaining extended services from community pharmacies. Faster access and convenience were the main features valued by patients, who also valued having sufficient time during consultations and a relaxed atmosphere for services such as blood pressure tests. For GPs, the main value derived from such services was freeing up their time to focus on more complex patients.

*“They’re [community pharmacies] more accessible and less formal”.* [F16, diabetes FG3]

*“Some of these medication reviews for asthma and COPD as well, you know, it will free some of our appointment time for us to, you know, deal with some other things”.* [M43, GP FG8]

## Discussion

This study used marketing theory to explore patients’, pharmacists’ and GPs’ views on utilisation and integration of community pharmacy services within the primary care pathway for patients with LTCs. Application of the 7Ps marketing mix theoretical framework has identified a number of key areas where developments could potentially increase patients’ (and GPs’) awareness of and demand for extended community pharmacy services, thus relieving some of the burden on general practice.

Findings from this study highlight important implications for policy and practice in relation to utilisation and integration of community pharmacy services within the primary care pathway for patients with LTCs. The potential for community pharmacies to moderate patient demands and reduce GP workload by providing services such as minor ailments and medication reviews has previously been recognised [27, 56–60]. However, this study has further highlighted strong support for community pharmacies to regularly provide routine check-ups/procedures (e.g. blood tests) for well managed LTCs.

Lack of access to medical records has been identified as a barrier to community pharmacists’ role expansion here, as in previous studies [61–65]. Having an integrated information system which enables community pharmacists’ read-write access to patient records may enhance communication with GPs and ensure safe provision of extended services. However, it would be important to consider indemnity and safeguarding against unauthorised access by non-healthcare staff. There have been some developments in this area in the UK with the implementation of the NHS Summary Care Record (SCR) in 2016 [66, 67]. However, the SCR provides read-only access and is limited to allergies, adverse reactions and medication history [68]. Another step towards integration could be to include community pharmacy services as part of care plans for patients with LTCs. Multidisciplinary care planning within primary care could help overcome common issues such as duplication of services [60, 69–71] and miscommunication between pharmacists and GPs [27, 64, 72, 73].

GPs’ willingness to collaborate with community pharmacists in the current study contrasted with previous findings [24, 58, 71, 74–76]. Nonetheless, this study strengthens the evidence for incentivising GPs to refer patients to community pharmacy services [19, 77, 78]. Remunerating joint working between community pharmacies and GP practices through the GP contractual framework could increase GP referrals to community pharmacies. Policy makers could also consider developing services with similar designs to influenza vaccinations and inhaler techniques as these services focus on a specific intervention. The current findings suggest that developing services with clear service specifications which focus on a particular problem could enhance the consistency and quality of service provision and encourage GP referrals. Increasing the consistency of service commissioning within and between different localities and regions may further enhance their uptake as it remains unclear which services are available to patients.

This study highlights the potential to promote community pharmacy services through national campaigns as patients are generally unware of what community pharmacies offer [22]. However, further research is needed to determine the most effective promotional strategies. The current findings also stress the importance of first-hand experience and word-of-mouth for enhancing the credibility of extended services to patients – this is something that any promotional strategies may wish to take account of. To ensure that extended services take equal priority to dispensing, reimbursement models should take account of the workload implications for community pharmacies. It is also important for pharmacies to ensure premises are suitable to deliver extended services and to fully utilise skill-mix by delegating more technical activities of medicines supply to pharmacy support staff [78].

To the authors’ knowledge, this is the first study to use marketing theory to understand how community pharmacy services could be better used and integrated within the primary care pathway for patients with LTCs. In addition, exploring the views of key stakeholders (patients, pharmacists, GPs) provided the breadth necessary to identify broad concepts influencing the use of community pharmacy services. Furthermore, this study was not limited to a specific service which widened the scope of the findings and implications. The application of the 7Ps marketing mix conceptualised key components influencing better use and integration of community pharmacy services within the primary care pathway. Hence, it led to the formation of a framework which can inform policy makers and future research in this area. Policy makers can use the ideas presented here from the 7Ps to develop strategies to enhance the development and integration of current/future community pharmacy services. Future research could apply this framework to evaluate the extent these 7P components could influence better use and integration of community pharmacy services within the primary care pathway.

A key limitation was the selection bias associated with the identification and recruitment of participants. Volunteers may have been more positive about the expanding role of community pharmacy. Future research could explore the opinions of a wider range of patients, pharmacists and GPs to compare/contrast findings in this study. Another limitation was having one researcher code the data. Nonetheless, one co-author co-facilitated each group, so were familiar with discussions and emerging themes, which added rigor to the process of data analysis and interpretation was reviewed and agreed between all authors. Any potential bias that could occur due to two of the authors being pharmacists was mitigated due to both co-authors being very experienced health services researchers, and one being a social scientist.

Whilst this study focused on community pharmacy services in England, findings could be tested/further explored in other countries with similar community pharmacy advancements such as the United States, Canada, Australia and New Zealand. However, differences in organisational and administrative context will need to be considered. Moreover, this study focussed on patients with respiratory conditions or type 2 diabetes as exemplar LTCs, and further research will need to establish whether the findings are applicable to other LTCs.

## Conclusion

This study used marketing theory to identify factors which could influence the utilisation and integration of community pharmacy services for patients with LTCs. In the main, these centred on appropriately distributing services within primary care, enhancing communication and incentivising joint working between community pharmacies and GP practices. Other factors involved enhancing the consistency and quality of community pharmacy services and strategically promoting community pharmacy services. Future research should evaluate the extent to which these factors could influence better use and integration of community pharmacy services within the primary care pathway and positively impact outcomes for patients with LTCs.

## Additional file

Additional file 1: Participant topic guides used for the focus groups. (DOCX 64 kb)

## Abbreviations

COPD: Chronic obstructive pulmonary disease
GP: General practitioner
LTC: Long-term condition
MUR: Medicines use review
NHS: National Health Service
NMS: New medicine service
SCR: Summary care record
