# Supplementary material for: Community pharmacy integration within the primary care pathway for people with long-term conditions: a focus group study of patients’, pharmacists’ and GPs’ experiences and expectations
Source: BMC Fam Pract. 2019 Feb 8;20:26. doi: 10.1186/s12875-019-0912-0 (PMC6368723; doi:10.1186/s12875-019-0912-0)
Supplement: Supplementary file 1 — Participant topic guides used for the focus groups. (DOCX 64 kb) [file 12875_2019_912_MOESM1_ESM.docx]

**Patient Interview Guide**

**Product (“service”)**

- Tell me what do you know about services a chemist can offer?

- Tell me what services do you think they should be offering for someone with a condition like yours?

- ***Prompts:*** *Are you aware chemists offer these services for* your condition *(offer a written list of services)? Would you use any of these services? Which ones?*
- ***Probes:*** *How do you feel about pharmacists taking more responsibilities in the management of your conditions? Why?*

- Tell me what do you know about the different services a **GP** practice can offer?

- Tell me what services do you think **GPs** should offer for someone with [state condition of particular FG]?

- Thinking about the chemist services we discussed earlier; would you prefer any of them to be performed by your GP instead? Which ones? Why? – Conversely, are there some that your GP currently offers, that you think could equally be offered by the chemist?

**Process**

**-** For those who visit chemists for services other than dispensing, could you describe how the process works? (I.e. how do you access services/how do you get recruited/ how does follow-up work?)

***Prompts****:* □ *busyness □ appointments □ waiting times*

- Now could you describe how the process works when using the GP practice for a consultation service in relation to your [condition]?

- ***Probe****: Is there anything you prefer about the way in which GP practices or chemists function?*

- For those who **do not** visit chemists for services other than dispensing, could you explain how you would want the process to work if you were to visit a chemist for such services?

**Price (Value)**

- What makes you/would make you choose to use your chemist as opposed to GP practice?

- ***Prompts:*** *□Contributing to their care □ Accessibility and convenience of the pharmacy □ Pharmacist availability*

*□ Pharmacist approachability*

- Thinking about some of the chemist services mentioned earlier in addition to the list provided, which do you feel are the most/least beneficial to you? Why?

- Would you be willing to pay for any of these services? If so, how much?

- ***Prompt:*** How about GP practice services?

**People**

- Tell me who do you get advice from about your medications when visiting the chemist?

- ***Probes:*** *How do they speak to you? How do they present themselves?*
- ***Prompts:*** *□ friendliness □ communication □ trustworthiness □ professionalism*

- Do you know who the pharmacist is? Do you know the other pharmacy staff, and how they differ?

- ***Probes:*** *Does knowing who the pharmacist is make any difference to you? How so/why not?*

- Are there any other healthcare professionals that you see for your [condition]?

- ***Probes:*** *Have they referred or recommend going to the pharmacy for something? If yes, what did they recommend? To what extent do they influence your decision to use chemists?*
- ***Prompt:*** *What about GPs?*

**Promotion**

- For those who visit chemists for services other than dispensing or are aware those services exist, can you remember when you first became aware that chemists provide these services for you?

- ***Probe:*** *Who told you?*
- ***Prompts****: □ friends/family □GPs □other pharmacists*

***-***  Is there anything that influences/would influence you to try new chemist services?

- ***Prompts****: □Posters □ leaflets □social media. What do you think of them? Do they work?*

- Is there anything else you think that can be done to advertise or promote chemist services?

**Physical evidence**

***-***  Which do you think about the environment of the chemist as a place to provide healthcare services for patients with long-term conditions?

- ***Prompts:*** *privacy and confidentiality of: chemists □ consultation rooms□*

**Place**

***-***  Can you describe how you feel about the accessibility of your chemist?

- ***Prompts****:* ***□****Opening hours □ Transport □ Parking □ Pharmacies: in a supermarket □ owned by large companies □ near GP surgeries*

**Final Statement**

The purpose of this study is to collect your views regarding community pharmacy services (chemists) for patients with long-term conditions. Is there **anything anyone would like to add** that you believe was not covered during this discussion?

**Pharmacist interview Guide**

**Product (“service”)**

***-*** Tell me what do you think patients know about services a pharmacy can offer?

***-*** Tell me are there any services that you think patients with asthma/ COPD/ diabetes want community pharmacies to offer them?

- ***Prompts:*** *Do you think they are aware pharmacies offer these services for* their conditions *(offer a written list of services)? Do you think they (would) use any of these services? Which ones?*
- ***Probes:*** *How do you think they feel about pharmacists taking more responsibilities in the management of their conditions? Why?*

***-*** Thinking about the pharmacy services we discussed earlier; do you think patients prefer any of them to be performed by their GP instead? Which ones? Why? – Conversely, are there some that GP currently offers, that they think could equally be offered by the community pharmacy?

***-*** Tell me what do you think **GPs** know about services a pharmacy can offer?

***-*** Tell me are there any services that you think **GPs** want community pharmacies to offer someone with a condition like asthma/ COPD/ diabetes?

- ***Prompt:*** *Do you think they are aware pharmacies offer these services for* these conditions *(refer to written list of services again)?*
- ***Probe:*** *How do you think they feel about pharmacists taking more responsibilities in the management of patients with conditions such as asthma/ COPD/ diabetes?*

***-*** Once again, referring to the pharmacy services we discussed earlier; do you think **GPs** prefer any of them to be performed by GP practices instead? Which ones? Why? – Conversely, are there some that GPs currently offer, that they think could equally be offered by the community pharmacy?

**Process**

***-*** Have you previously encountered a patient with asthma/COPD/diabetes who used the pharmacy for a healthcare service other than dispensing? If so, could you describe how the process works? (I.e. how do patients access services/how do you recruit them/ how do you follow-up?)

- ***Prompts****:* □ *busyness □ appointments □ waiting times*

***-*** How do you think **GPs** feel about patients with these conditions using the pharmacy for services other than dispensing?

- ***Probe:*** *What do you think they like/dislike about patients using the pharmacy for such services?*
- ***Prompts****: □ advice given to patients □ reducing workload □ conflicting with their roles*

**Price (Value)**

***-*** Explain to me why do you think patients (would) choose to use the pharmacy as opposed to GP practice?

- ***Prompts:*** *□Contributing to their care □ Accessibility and convenience of the pharmacy □ Pharmacist availability*

*□ Pharmacist approachability*

***-*** Thinking about some of the pharmacy services mentioned earlier in addition to the list provided, which services do you think are most/least beneficial for patients with long-term conditions?

- ***Probes:*** *Do you think patients/GPs would agree?*

***-*** In terms of **GPs,** do you think they would be willing to recommend any of these services to their patients? If so, which services do you think they are most willing to recommend? Which services are they least willing to recommend? Why?

- Would patients be willing to pay for any of these services? If so, how much?

- ***Probe:*** *How about GP practice services?*

**People**

***-***  Tell me do you think pharmacists and pharmacy staff are in a position to be able to deliver these services well? Why/why not?

- ***Prompts:*** *□Workload □Corporate pressure □ Motivation □ Staff □Training □ Standards*

***-***  Describe how you think pharmacy staff could communicate effectively/more effectively with patients?

- ***Prompts:*** *□ friendliness □ communication □ trustworthiness □ professionalism*

***-***  Do you know the GP(s) in your local area? How often do you communicate with them?

- ***Probes:*** *How do they speak to you? How do they present themselves? How do you think you could communicate more effectively with them? Does knowing the GP make any difference to you/patient care? How so/why not?*

**Promotion**

***-*** If you are familiar with patients who visit pharmacies for services other than dispensing or are aware those services exist, how do you think they first became aware that pharmacies provide these services for them?

- ***Probe:*** *Who do you think tells them?*
- ***Prompts****: □ friends/family □GPs □other pharmacists*

***-***  Is there anything that you think influences/would influence patients to try new pharmacy services?

- ***Prompts****: □Posters □ leaflets □social media. What do you think of them? Do they work?*

***-*** Is there anything that you think influences/would influence **GPs** to recommend new pharmacy services?

***-***  Explain to me how do you think community pharmacy services should be promoted to patients?

- ***Probe****: Who do you think should be responsible for this?*

**Physical evidence**

***-***  Which do you think about the environment of the pharmacy as a place to provide healthcare services for patients with long-term conditions?

- ***Prompts:*** *privacy and confidentiality of: community pharmacies□ your consultation room□*

**Place**

***-***  Explain to me how you think patients feel about the accessibility of community pharmacies?

- ***Prompts****:* ***□****Opening hours □ Transport □ Parking □ Pharmacies: in a supermarket □ owned by large companies □ near GP surgeries*

***-*** Do you think **GPs** share the same view? How so?

**Final Statement**

The purpose of this study is to collect your views regarding community pharmacy services for patients with long-term conditions. Is there **anything anyone would like to add** that you believe was not covered during this discussion?

**GP interview Guide**

**Product (“service”)**

- Tell me what do you know about services a community pharmacy can offer?

- Tell me what services do you think they should be offering for someone with a condition like asthma/COPD/diabetes?

- ***Prompts:*** *Are you aware community pharmacies offer these services for* these conditions *(offer a written list of services)? Would you recommend patients to use any of these services? Which ones?*
- ***Probes:*** *How do you feel about pharmacists taking more responsibilities in the management of patients with these conditions? Why?*

- Thinking about the pharmacy services we discussed earlier; would you prefer any of them to be performed by GPs instead? Which ones? Why? – Conversely, are there some services that GPs currently offer, that you think could equally be offered by the community pharmacy?

**Process**

**-** How do you feel about patients using the pharmacy for services other than dispensing?

- ***Probe:*** What do you like/not like about patients using the pharmacy for such a service?
- ***Prompts****:* □ *reducing your workload □ conflicting with your roles □ advice given*

**Price (Value)**

- Thinking about some of the pharmacy services mentioned earlier in addition to the list provided, which do you feel are the most/least beneficial to your patients? Why?

- Do you think patients would be willing to pay for any of these services? If so, how much?

- ***Prompt:*** How about GP practice services?

**People**

- Do you know the community pharmacist(s) in your local area? How often do you communicate with them?

- ***Probes:*** *How do they speak to you? How do they present themselves?*
- ***Prompts:*** *□ friendliness □ communication □ trustworthiness □ professionalism*

- Does knowing who the pharmacist is make any difference to you? How so/why not?

- Have you referred or recommend any of your patients to go to the pharmacy for something? If yes, what did you recommend? Did they take the recommendation?

**Promotion**

- For those who have recommended pharmacy services other than dispensing or are aware those services exist, can you remember when you first became aware that pharmacies provide these services?

- ***Probe:*** *Who told you?*
- ***Prompts****: □ friends/family □other GPs □pharmacists*

***-***  Is there anything that influences/would influence you to recommend new pharmacy services to your patients?

- ***Prompts****: □Posters □ leaflets □social media. What do you think of them? Do they work?*

- Explain to me how do you think community pharmacy services should be promoted to patients?

- ***Probe:*** *Who should be responsible for this?*

**Physical evidence**

***-***  Which do you think about the environment of the pharmacy as a place to provide healthcare services for patients with long-term conditions?

- ***Prompts:*** *privacy and confidentiality of: community pharmacies □ consultation rooms□*

**Place**

***-***  Explain to me how you think patients feel about the accessibility of community pharmacies?

***Prompts****:* ***□****Opening hours □ Transport □ Parking □ Pharmacies: in a supermarket □ owned by large companies □ near GP surgeries*

**Final Statement**

The purpose of this study is to collect your views regarding community pharmacy services for patients with long-term conditions. Is there **anything anyone would like to add** that you believe was not covered during this discussion?
